# Multiple Campylobacter Genomes Sequenced

**DOI:** 10.1371/journal.pbio.0030040

**Published:** 2005-01-04

In 1995, the first complete bacterial genome sequence was published. Now, nearly 200 bacterial genomes have been completed, and a new one hits the scientific press most weeks. This burgeoning industry is not just scientific “stamp collecting,” however. Having all these genome sequences may provide useful clues about why some bacteria cause human disease, how to control their spread, and how to treat the infections caused by them. By comparing genome sequences, scientists can learn much more about what makes a bacteria tick than they can learn from a single sequence.

Derrick Fouts and his colleagues have taken this comparative approach with Campylobacter. Infection with a Campylobacter species is one of the most common causes of human bacterial gastroenteritis. In the US, 15 out of every 100,000 people are diagnosed with campylobacteriosis every year, and with many cases going unreported, up to 0.5% of the general population may unknowingly harbor Campylobacter in their gut annually. Diarrhea, cramps, abdominal pain, and fever develop within 2–5 days of picking up a pathogenic Campylobacter species, and in most people, the illness lasts for 7–10 days. But the infection can sometimes be fatal, and some individuals develop Guillain-Barré syndrome, in which the nerves that join the spinal cord and brain to the rest of the body are damaged, sometimes permanently.

Campylobacteriosis is usually caused by C. jejuni, a spiral-shaped bacterium normally found in cattle, swine, and birds, where it causes no problems. But the illness can also be caused by C. coli (also found in cattle, swine, and birds), C. upsaliensis (found in cats and dogs), and C. lari (present in seabirds in particular). Disease-causing bacteria generally get into people via contaminated food, often undercooked or poorly handled poultry, although contact with contaminated water, livestock, or household pets can also cause disease.[Fig pbio-0030040-g001]

**Figure pbio-0030040-g001:**
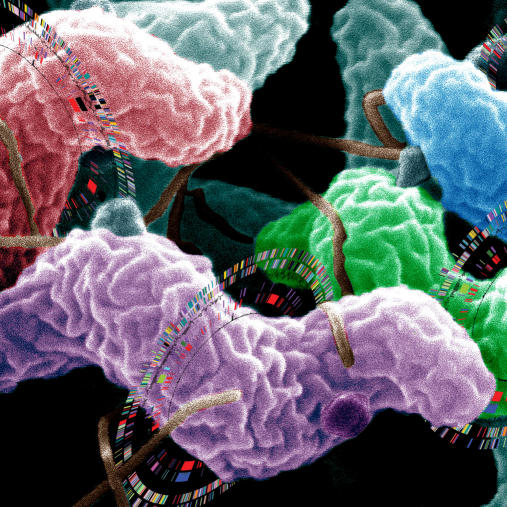
Genome sequencing and comparison of four species of Campylobacter

In 2000, C. jejuni was the first food-borne pathogen to be completely sequenced, but we still know little about how Campylobacter species cause disease. In their search for clues, Derrick Fouts and coworkers have completely sequenced the genome of C. jejuni strain RM1221 (isolated from a chicken carcass) and compared it with the previously sequenced C. jejuni strain NCTC 11168 and with the unfinished sequences of C. coli strain RM2228 (a multi-drug-resistant chicken isolate), C. lari strain RM2100 (a clinical isolate), and C. upsaliensis strain RM3195 (taken from a patient with Guillain-Barré syndrome).

The researchers describe numerous differences and similarities between these different Campylobacter strains and species. For example, there are major structural differences between the genomes caused by the insertion of new stretches of DNA. Some of these pieces of DNA may carry genes that improve bacterial virulence or fitness, so their presence could help to explain the different biological behaviors of these strains. There are also major variations in the genes responsible for synthesis of molecules that are important for the interaction of Campylobacter with the environment. Such differences could underlie the host specificity of the different species.

Differences between the Campylobacter species in genes that are likely to be involved in aspects of bacterial virulence, such as adherence, motility, and toxin formation, are all detailed by Fouts et al., who also describe a new putative Campylobacter virulence locus. Further work is needed to relate these genomic differences to functional differences, but this detailed comparative genomic analysis provides the core blueprint for this important family of human pathogens. And in doing so, it lays the foundation for the development of new ways to monitor and control Campylobacter in the food chain and in human infection.

